# Supplementary material for: AntiDMPpred: a web service for identifying anti-diabetic peptides
Source: PeerJ. 2022 Jun 14;10:e13581. doi: 10.7717/peerj.13581 (PMC9205309; doi:10.7717/peerj.13581)
Supplement: Supplemental Information 1 — Parameters to be optimized in the machine learning methods and feature selection. [file peerj-10-13581-s001.docx]

**Supplementary Information for**

**AntiDMPpred: a web service for identifying anti-diabetic peptides**

Xue Chen^1^, Jian Huang^2^, Bifang He^1^

^1^Medical College, Guizhou University, Guiyang 550025, Guizhou, China

^2^School of Life Science and Technology, University of Electronic Science and Technology of China, Chengdu 6173001, Sichuan, China

Corresponding Author:

Bifang He

Huaxi District, Guiyang, Guizhou, 550025, China

Email address: bfhe@gzu.edu.cn

Jian Huang

No.2006, Xiyuan Ave, West Hi-Tech Zone, Chengdu 6173001, Sichuan, China

Email address: hj@uestc.edu.cn

Supplementary Table 1 Parameters to be optimized in machine learning methods

| **Machine learning method** | **Parameter to be optimized** |
| --- | --- |
| Random Forest (RF) | Number of trees |
| K-Nearest Neighbor (KNN) | k |
| LinearSVM | c |
| RBFSVM | c, g |

Supplementary Table 2 Parameters to be optimized in the feature selection

| **Feature selection method** | **Parameter to be optimized** |
| --- | --- |
| F-score (Fscore) | Feature number |
| K-means (Kmeans) | Cluster number |
| Lasso | alpha, Feature number |
| Pearson correlation (Pearson) | Feature number |
| Spearman correlation (Spearman) | Feature number |
| Student's *t*-test (Ttest) | Feature number |

Supplementary Table 3 Performance comparison of data augmentation with original dataset

| **Feature scoring method** | **Machine learning method** | **Dataset** | **Accuracy (%)** | **Sensitivity (%)** | **Specificity (%)** | **Precision (%)** | **MCC** | **F1** | **AUCROC** |
| --- | --- | --- | --- | --- | --- | --- | --- | --- | --- |
| Pearson | RF | original | 77.12 | 80.51 | 73.73 | 75.40 | 0.5436 | 0.7787 | 0.8193 |
|  |  | 100% | 75.00 | 77.97 | 72.03 | 73.60 | 0.5009 | 0.7572 | 0.8138 |
|  |  | 200% | 73.52 | 77.12 | 69.92 | 71.94 | 0.4716 | 0.7444 | 0.8109 |
|  |  | 300% | 75.85 | 77.97 | 73.73 | 77.33 | 0.5218 | 0.7549 | 0.8314 |
|  | KNN | original | 72.46 | 76.69 | 68.22 | 70.70 | 0.4508 | 0.7358 | / |
|  |  | 100% | 70.13 | 72.03 | 68.22 | 69.39 | 0.4028 | 0.7069 | / |
|  |  | 200% | 72.03 | 73.73 | 70.34 | 71.31 | 0.4409 | 0.7250 | / |
|  |  | 300% | 72.46 | 74.15 | 70.76 | 73.57 | 0.4537 | 0.7212 | / |
|  | LinearSVM | original | 67.58 | 70.76 | 64.41 | 66.53 | 0.3524 | 0.6858 | 0.7237 |
|  |  | 100% | 67.58 | 69.07 | 66.10 | 67.08 | 0.3519 | 0.6806 | 0.7350 |
|  |  | 200% | 69.28 | 72.03 | 66.53 | 68.27 | 0.3862 | 0.7010 | 0.7395 |
|  |  | 300% | 65.47 | 68.64 | 62.29 | 66.82 | 0.3143 | 0.6447 | 0.7265 |
|  | rbfSVM | original | 69.07 | 68.64 | 69.49 | 69.23 | 0.3814 | 0.6894 | 0.7374 |
|  |  | 100% | 68.86 | 67.37 | 70.34 | 69.43 | 0.3773 | 0.6839 | 0.7160 |
|  |  | 200% | 68.43 | 69.49 | 67.37 | 68.05 | 0.3687 | 0.6876 | 0.7195 |
|  |  | 300% | 66.95 | 68.64 | 65.25 | 67.84 | 0.3435 | 0.6652 | 0.6964 |

Supplementary Table 4 Description of each peptide database

| **Database** | **The total number of peptides** | **The number of peptides after processing** | **Overlapping peptide with BioDADpep** | **Frequency** |
| --- | --- | --- | --- | --- |
| AVPdb | 2059 | 2025 | 0 | 0 |
| BioPepDB | 4807 | 3717 | 19 | 0.51% |
| CAMPr3 | 8225 | 3985 | 0 | 0 |
| CancerPPD | 3142 | 2656 | 0 | 0 |
| CPPsite | 1564 | 1494 | 0 | 0 |
| DRAMP | 28023 | 23433 | 0 | 0 |
| HIPdb | 981 | 950 | 0 | 0 |
| SATPdb | 16590 | 15232 | 20 | 0.13% |
| THPdb | 894 | 78 | 0 | 0 |
| UniProt | 4164 | 4122 | 0 | 0 |
| Average | | | | 0.068% |
